# Genotypic Analysis of *Enterobius vermicularis* (Rhabditida: Oxyuridae, Linnaeus, 1758) Among Infected Individuals in Bulgaria: A First Phylogenetic Study

**DOI:** 10.3390/ijms27042020

**Published:** 2026-02-20

**Authors:** Eleonora Kaneva, Reneta Dimitrova, Nina Tsvetkova, Rumen Harizanov, Desislava Velcheva, Aleksandra Ivanova, Mihaela Videnova, Raina Borisova, Maria Pavlova, Diana Jordanova, Ivailo Alexiev

**Affiliations:** 1 Department of Parasitology and Tropical Medicine, National Centre of Infectious and Parasitic Diseases, 26 Yanko Sakazov Blvd., 1504 Sofia, Bulgaria; kaneva@ncipd.org (E.K.); tsvetkova@ncipd.org (N.T.); aleksandra.ivanova@ncipd.org (A.I.); mvidenova@ncipd.org (M.V.); rainaborisova@ncipd.org (R.B.); 2 Department of Virology, National Centre of Infectious and Parasitic Diseases, 44A Gen. Stoletov Blvd., 1233 Sofia, Bulgaria; naydenova.reneta@gmail.com (R.D.); ivoalexiev@yahoo.com (I.A.); 3 Medical Diagnostic Laboratory “Cibalab”, 83 “Gyueshevo” Str., 1379 Sofia, Bulgaria; desislavasv@abv.bg; 4 Department of Microbiology, National Centre of Infectious and Parasitic Diseases, 26 Yanko Sakazov Blvd., 1504 Sofia, Bulgaria; mimipavlovaa@gmail.com; 5 Medical Diagnostic Laboratory “Ramus”, 26 “Capitan Dimitar Spisarevski” Str., 1592 Sofia, Bulgaria; jordanova.1947@abv.bg

**Keywords:** *Enterobius vermicularis*, enterobiasis, molecular epidemiology, *cox1* gene, genotype B, phylogenetic analysis

## Abstract

Enterobiasis, caused by the nematode *Enterobius vermicularis*, remains a widespread public health issue, yet data regarding its genetic structure in Southeast Europe are scarce. This study presents the first molecular and phylogenetic characterization of *E. vermicularis* isolates from Bulgaria. Between 2022 and 2025, perianal tape test samples were collected from 128 individuals (92.2% of whom were children) with enterobiasis from 17 regions of the country. Molecular identification was performed via nested PCR targeting a 324 bp fragment of the mitochondrial cytochrome c oxidase subunit 1 (*cox1*) gene, followed by Sanger sequencing. Phylogenetic relationships were analyzed using Maximum Likelihood (IQ-TREE), and population genetic indices were calculated using DnaSP v6. Phylogenetic analysis revealed that all 128 Bulgarian isolates belong to genotype B, clustering closely with sequences from other European and Asian countries. Genetic diversity analysis showed remarkably low variation, with a haplotype diversity (Hd) of 0.1507 ± 0.0416 and a nucleotide diversity (π) of 0.00082 ± 0.00015. Among the 11 identified haplotypes, a single dominant haplotype (Hap_1) accounted for 92.2% of all samples and was distributed across all sampled geographic regions. Tajima’s D was significantly negative (−2.314, < 0.05), suggesting a recent population expansion or purifying selection. The dominance of genotype B and the extremely low genetic diversity suggest a recent introduction or clonal expansion of *E. vermicularis* in Bulgaria. These findings provide essential baseline data for monitoring transmission dynamics and implementing effective control strategies in the Balkan region.

## 1. Introduction

*Enterobius vermicularis* (Rhabditida: Oxyuridae, Linnaeus, 1758), (*E. vermicularis,* synonym: *Oxyuris vermicularis*) is a widespread small, filamentous nematode with a simple, direct life cycle that takes place in the lumen of the human gastrointestinal tract [1]. Human infection occurs via the oral route or inhalation. Frequent repeat invasions are observed, resulting from significant environmental contamination with parasitic stages [2]. Due to the lack of an intermediate host, this parasite is characterized by rapid transmission [3] and it is the only nematode of the family *Oxyuridae* that infects humans and causes enterobiasis. Most *Enterobius vermicularis* infections are asymptomatic, without the appearance of any clinical signs, which is the cause for the hidden spread of the disease and the infection of contacts of the invaded persons—most often children in organized institutions and household members.

*E. vermicularis* is a parasite distributed mainly among humans and primates, as the co-evolutionary path seems to be the most acceptable hypothesis for explaining the origin of human pinworms [4]. Fossil findings confirm its coexistence alongside humans dating back to thousands of years [5]. Host specificity is exceptional in pinworms, as each species of nematode is found in a specific host [6], and besides humans, this parasite is also found in most genera of the order Primates. In some pre-historic cultures, *E. vermicularis* reached a very high prevalence, as established in coprolites containing eggs of this helminth [7].

Despite the wide distribution and the ancient origin of *E. vermicularis* being well-established, genetic studies of this parasite are limited. The main diagnostic method, microscopy of a perianal imprint taken with transparent adhesive tape, has low sensitivity. This leads to many undiagnosed cases and makes controlling enterobiasis difficult [1]. In recent years, therefore, molecular biological methods have been developed that are highly sensitive and specific, allowing for further phylogenetic studies. These studies are of great importance for determining the genetic characteristics of this parasite.

Molecular and phylogenetic studies of *E. vermicularis* fall into two categories of research: those based on ribosomal DNA and those based on mitochondrial DNA. The complete, highly conserved 5S ribosomal intergenic region of the pinworm is approximately 800 bp [8], and there are several copies of it per organism. A method for the molecular detection of pinworms in archaeology was developed using nested PCR targeting the *E. vermicularis* 5S rRNA spacer region, which strongly outperforms microscopic analysis [9,10]. The rDNA sequences of the small subunit were used to construct the molecular phylogeny of Nematoda [11,12], which did not yet include *E. vermicularis*. Therefore, Zelck and colleagues analyzed 18S rDNA sequences of *E. vermicularis*, along with nematode sequences published by Nadler in GenBank (NCBI) [13]. After analysis, the placement of *E. vermicularis* in the order Oxyuridae was confirmed. For this study, Zelck and his colleagues amplified 18S rDNA for diagnostic purposes using *Enterobius*-specific primers Ev18S.F1 and Ev18S.R1. They sequenced DNA from the small ribosomal subunit (18S rDNA, 1716 bp), the first and second internal transcribed spacer regions (ITS1 and ITS2, 1073 bp), 5.8S rDNA (159 bp), and a 78-bp fragment of the large ribosomal subunit (28S). The molecular analysis performed here did not reveal sufficient diversity in the studied genes to distinguish *E. vermicularis* isolates [13]. This directs research on the genetic diversity of the parasite toward the mitochondrial genome. The complete mitochondrial genome sequence of the human pinworm, *E. vermicularis* (Oxyurida: Nematoda), was determined in 2009. This sequence was then used to infer its phylogenetic relationship to other major groups of chromadorean nematodes [14]. In recent years, a series of studies have been performed in which molecular and genotyping studies using mitochondrial markers (mtDNA) were executed to determine the genetic variability of *E. vermicularis*. Various phylogenetic studies from Asian and European countries have identified three populations or subtypes of the parasite: Genotypes A and B were isolated from humans and primates, such as chimpanzees, whereas genotype C has only been reported in chimpanzees. As of yet, no studies have found genotype C *E. vermicularis* in humans [15].

According to published scientific reports on this issue, genotype B is the predominant pinworm genotype in Europe. However, such data are not yet available for Bulgaria. Therefore, our study aims to perform genetic and phylogenetic analysis of *E. vermicularis* in samples obtained from infected patients in Bulgaria in order to determine the prevalent genotypes/haplotypes and genetic differences in this parasite in the country.

## 2. Results

### 2.1. Epidemiological and Demographic Characteristics of the Study Population

The study cohort comprised 128 individuals with confirmed *E. vermicularis* infection from 17 Bulgarian administrative regions. The population was predominantly pediatric, with adults representing only 7.8%. The gender distribution was approximately balanced, including 66 females and 62 males (Table 1).

The highest prevalence among pediatric cases occurred in the 4–6 age group, followed by the 7–9, 10–12, and 1–3 age groups. Notably, no cases were identified in the 13–15 age group, and only two cases were recorded in the 16–18 age group.

Geographically, the majority of cases were concentrated in Sofia City, followed by Sofia District and 15 other regions, which collectively contributed 36.8% of cases. Statistical analysis revealed no significant association between gender and age distribution (*χ*^2^ = 3.47, *df* = 5, *p* = 0.628) or between geographic origin and demographic variables (Fisher’s exact test, *p* = 0.747).

### 2.2. Molecular Identification and Genotyping

Molecular characterization of *E. vermicularis* was successfully achieved using the mitochondrial cytochrome c oxidase subunit 1 (*cox1*) gene. A 324-bp fragment was amplified and sequenced from all 128 clinical samples collected across Bulgaria. This demonstrated the reliability of this molecular approach for identifying parasites from adhesive tape specimens.

### 2.3. Phylogenetic Analysis and Genotype Distribution

**Figure 1:**
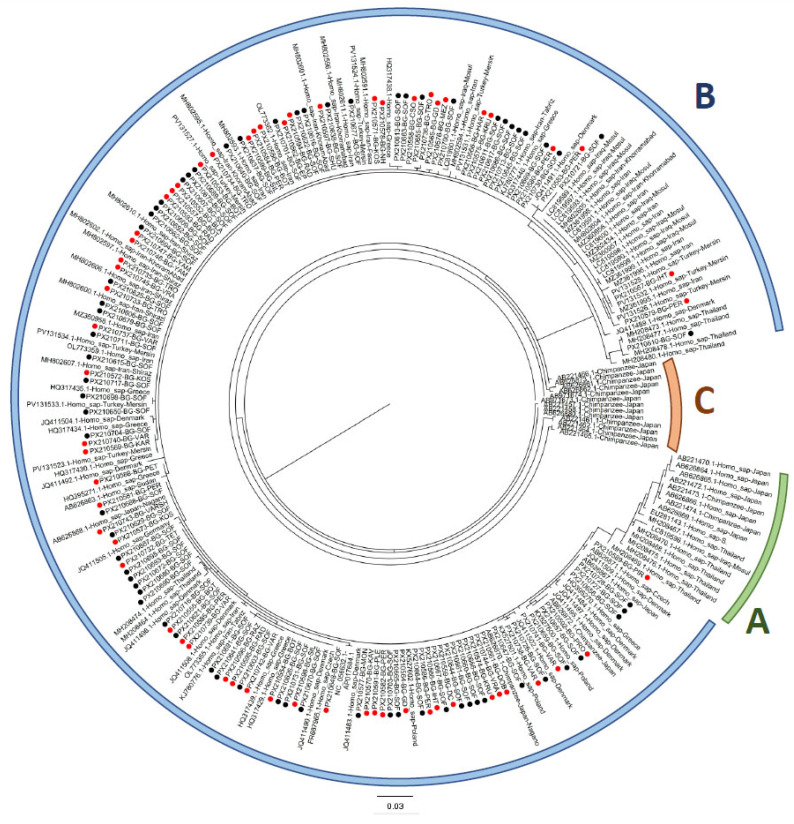
Maximum-likelihood phylogenetic tree of *E. vermicularis cox1* sequences. Black circles indicate sequences from Sofia, and red circles indicate sequences from other Bulgarian regions. Colored arcs denote the three major genotypes (A, B, and C). All Bulgarian sequences cluster within genotype B. Region abbreviations used in the sequence identifiers are defined in the text.

Phylogenetic reconstruction using the maximum likelihood (ML) method revealed that all 128 Bulgarian sequences belonged exclusively to genotype B. No representatives of genotypes A or C were detected (Figure 1). Within genotype B, the Bulgarian sequences exhibited high genetic homogeneity, with 92.2% (118/128) belonging to a single dominant haplotype. While the phylogenetic reconstruction placed these sequences in proximity to various global isolates, including those from Iran, Greece, and Denmark, it should be noted that the internal nodes within genotype B lacked robust bootstrap support due to limited sequence variation. Consequently, these associations represent close genetic similarities rather than clearly defined evolutionary lineages.

Geographic analysis within Bulgaria revealed no clear phylogeographic structure. Sequences from the same administrative region were distributed across multiple clades within genotype B. This suggests either multiple introduction events or extensive gene flow within the country. For example, the PX210610-BG-SOF sequence from Sofia clustered with Thai sequences at the root of genotype B. In contrast, the PX210579-BG-PER (Pernik) and PX210567-BG-IHT (Ihtiman) sequences formed a distinct cluster with Iranian and Turkish isolates. This indicates that there may have been multiple introduction pathways into Bulgaria.

### 2.4. Genetic Diversity and Population Structure

The PCR amplification yielded products of 390 bp and 379 bp, which were subsequently trimmed to remove primer sequences and terminal regions, resulting in a uniform 324 bp alignment for all 128 Bulgarian *E. vermicularis* sequences. Genetic diversity analysis revealed exceptionally low variation within the population (Table 2 and Table 3). After the exclusion of sites containing gaps and missing data to ensure consistent phylogenetic analysis, a total of 292 positions were analyzed from the 324 bp alignment. Only 13 sites (4.5%) showed polymorphism, indicating remarkable sequence conservation. Haplotype analysis identified 11 distinct haplotypes with an overall haplotype diversity (Hd) of 0.1507 ± 0.0416, which is substantially lower than values reported from other geographic regions. A single haplotype (Hap_1), encompassing 118 sequences (92.2%), dominated the population structure and was uniformly distributed across all 17 sampled administrative regions (Table 2). The remaining ten haplotypes (Hap_2–Hap_11) were all singletons, each represented by a single sequence (0.78% each). These rare variants showed no geographic clustering: Hap_2 was identified in Ihtiman; Hap_3 and Hap_4 in Pernik; Hap_5 in Pirdop; Hap_6 through Hap_10 in Sofia; and Hap_11 in Troyan. This distribution pattern suggests that rare variants arise sporadically through mutation rather than geographic isolation.

The remaining ten haplotypes were all singletons, each represented by a single sequence. These rare variants showed no geographic clustering. Hap_2 (PX210567-BG-IHT) originated in Ihtiman; Hap_3 (PX210579-BG-PER) and Hap_4 (PX210583-BG-PER) originated in Pernik; Hap_5 (PX210589-BG-PIR) originated in Pirdop; and Hap_6 through Hap_10 (PX210610-BG-SOF; PX210687-BG-SOF; PX210698-BG-SOF; PX210700-BG-SOF; PX210721-BG-SOF) originated in Sofia. Hap_11 (PX210736-BG-TRO) originated in Troyan. This distribution pattern suggests that rare variants arise sporadically through mutation rather than geographic isolation.

Nucleotide diversity (π = 0.00082 ± 0.00015) was correspondingly low, which further confirmed the limited genetic variation. The distribution of polymorphic sites revealed that all variable positions represented transitions (9/13) rather than transversions (4/13), consistent with neutral evolution patterns in mitochondrial DNA (Table 3).

The Tajima’s D test yielded a significantly negative value, indicating a deviation from neutral evolution. Together with the star-like topology of the haplotype network, characterized by a single dominant haplotype and multiple singleton variants, this negative value suggests a pattern consistent with recent population expansion or a selective sweep. The presence of ten singleton haplotypes (90.9% of all haplotypes) relative to a single high-frequency haplotype supports the demographic expansion model, in which newly arisen mutations have not yet increased in frequency due to genetic drift. Furthermore, the absence of intermediate-frequency haplotypes and the lack of clear geographic structuring are in line with a scenario of recent introduction followed by rapid expansion, consistent with patterns of increased human mobility and globalization observed in recent decades.

## 3. Discussion

This study represents the first comprehensive molecular characterization of *E. vermicularis* in Bulgaria and Southeast Europe, revealing remarkably low genetic diversity (Hd = 0.1507) compared to global populations. The exclusive presence of genotype B aligns with previous European studies [16,17,18], confirming the continental distribution pattern of this genotype. However, the extremely low haplotype diversity and dominance of a single haplotype (92.2%) distinguishes the Bulgarian population from other European populations where multiple haplotypes typically co-exist at more balanced frequencies.

### 3.1. Population Genetics, Phylogeography, and Molecular Evolution

The significantly negative Tajima’s D value (−2.314, < 0.05) combined with the star-like haplotype network topology strongly suggests recent population expansion following a founder event. This demographic scenario is consistent with either recent introduction of *E. vermicularis* into Bulgaria or a severe population bottleneck followed by rapid expansion. Similar patterns have been observed in other parasitic nematodes following geographical expansion [19]. The predominance of singleton haplotypes (10 out of 11) surrounding a single dominant variant is a classic signature of recent population expansion from a small founding population [20].

The lack of geographic structuring despite sampling from 17 administrative regions indicates efficient transmission networks throughout the country, possibly facilitated by human migration, institutional settings, and modern transportation infrastructure. This finding contrasts with studies from larger geographic areas where significant population structuring has been observed [21]. The concentration of cases in Sofia City (52.3%) reflects typical urban transmission patterns, consistent with higher population density and increased contact rates in childcare facilities [22].

These population genetic patterns must be interpreted within the broader context of phylogeographic relationships and potential introduction pathways. Phylogenetic analysis revealed complex clustering with global isolates, deviating from standard isolation-by-distance models. Our results indicate that Bulgarian sequences share high genetic similarity with various global isolates, particularly those from Iran (27.3%), Greece (15.6%), and Denmark (11.7%). While the phylogenetic placement of certain sequences, such as the positioning of PX210610-BG-SOF in proximity to Thai isolates or PX210579-BG-PER and PX210567-BG-IHT within clades containing Iran-Turkish sequences could suggest multiple introduction events, these observations should be interpreted with caution. Due to the limited sequence variation and low bootstrap support for internal nodes within Genotype B, these groupings likely reflect close genetic affinities rather than robustly defined evolutionary lineages. Despite these potential diverse links, Bulgarian populations maintain remarkably low overall genetic diversity, dominated by a single widespread haplotype. This suggests restricted cross-border transmission or unique introduction histories. The strategic position of Bulgaria at the crossroads of Europe and Asia likely drives these heterogeneous phylogenetic associations [23,24].

The molecular evolutionary patterns observed in Bulgarian *E. vermicularis* populations provide further insights into the dynamics of parasite establishment and spread. The exclusive presence of genotype B in all 128 Bulgarian isolates confirms previous reports of the dominance of this genotype in Europe [17,18]. However, the complete absence of genotypes A and C, which have been found in other European countries, is noteworthy. Genotype A has been reported from Japan in human samples [25], while genotype C remains restricted to non-human primates.

The low nucleotide diversity (π = 0.00082) observed in Bulgarian populations is substantially lower than values reported from Denmark (π = 0.0032) [17] and Iran (π = 0.0045) [26]. The predominance of transitions over transversions (9:4 ratio) in polymorphic sites aligns with typical mitochondrial DNA evolution patterns [27]. This transition bias, combined with the low overall diversity, suggests that most mutations are recent and have not undergone significant selection. The integration of population genetic, phylogeographic, and molecular evolutionary data thus paints a coherent picture of *E. vermicularis* in Bulgaria as a recently established or bottlenecked population derived from multiple geographic sources, yet exhibiting limited subsequent diversification.

### 3.2. Epidemiological Implications and Transmission Dynamics

The age distribution of infected individuals, with 92.2% being children and peak prevalence in the 4–6 years age group (41.4%), mirrors global epidemiological patterns [28]. This concentration among preschool-aged children reflects behavioral factors including poor hygiene practices, close contact in institutional settings, and hand-to-mouth behavior typical of this age group [29]. The absence of cases in the 13–15 years age bracket suggests improved hygiene practices with age or possible acquired immunity [30].

The balanced gender distribution (51.6% females, 48.4% males) with no significant association between sex and age indicates equal exposure risk, contrasting with some studies reporting gender-based differences in infection rates [31]. This equality likely reflects similar exposure patterns in Bulgarian childcare settings and environments.

The successful amplification of *cox1* gene from all 128 clinical samples demonstrates the reliability of our molecular approach, addressing limitations of traditional microscopy which has notoriously low sensitivity [1]. Our nested PCR protocol, modified from Piperaki et al. (2011) [16], proved particularly effective for detecting *E. vermicularis* DNA from adhesive tape samples, overcoming challenges associated with PCR inhibitors in clinical specimens [32].

### 3.3. Public Health Significance, Control Implications, and Future Research Directions

The dominance of a single haplotype suggests that current *E. vermicularis* transmission in Bulgaria may originate from a limited number of sources, presenting opportunities for targeted intervention strategies. The low genetic diversity also has implications for anthelmintic resistance monitoring. Although resistance to commonly used drugs (mebendazole, albendazole) has been reported in other nematodes [33], the limited genetic variation in Bulgarian *E. vermicularis* populations suggests that resistance, if it emerges, might spread rapidly through the population due to limited standing genetic variation [34].

Our findings provide crucial baseline data for future monitoring of parasite evolution and transmission dynamics. The establishment of this genetic baseline is particularly important given increasing human mobility and globalization, which may introduce new genetic variants [19]. Regular molecular surveillance could detect new introductions, monitoring of changes in genetic diversity, and identification of emerging drug resistance markers.

Building upon these public health implications, several avenues warrant further investigation to enhance our understanding and improve control strategies. Temporal sampling would reveal whether genetic diversity is increasing through mutation accumulation or new introductions [35]. Analysis of additional genetic markers, particularly nuclear genes, could provide higher resolution of population structure and reveal potential hybridization events [36]. Investigation of functional genes related to drug resistance would have direct clinical applications [37].

Cross-border studies incorporating samples from neighboring Balkan countries would elucidate regional transmission patterns and pathogen migration routes. Given the phylogenetic associations with Iranian and Turkish isolates, investigating potential transmission corridors along historical trade and pathogen migration routes could reveal introduction pathways [38]. Such comprehensive regional approaches would not only advance our scientific understanding but also inform evidence-based public health interventions across Southeast Europe.

### 3.4. Methodological Considerations and Innovations

The conservative treatment of ambiguous nucleotide positions as missing data represents a methodological strength, ensuring that only high-confidence variations contribute to our analyses. While this approach may slightly underestimate diversity, it prevents artificial inflation of genetic variation caused by sequencing artifacts [39]. The successful application of multiple complementary analytical approaches, phylogenetic reconstruction, haplotype network analysis, and population genetic statistics, provides robust support for our conclusions.

The use of freeze–thaw cycles to optimize DNA extraction from resistant *E. vermicularis* eggs represents a simple but effective innovation that could benefit other studies of helminth genetics [40]. This pre-treatment step, combined with extended proteinase K digestion, significantly improved DNA yield from the notoriously resistant egg shells.

### 3.5. Study Limitations

Several limitations of the study should be acknowledged. First, the cross-sectional design does not allow for temporal tracking of genetic changes within populations. Second, ambiguous nucleotide positions were conservatively coded as missing data, which may slightly underestimate genetic diversity, although the overall impact is expected to be minimal given the low diversity observed. Third, the study focused exclusively on the *cox1* gene; inclusion of additional genetic markers could provide a more comprehensive understanding of population structure and evolutionary dynamics. Finally, samples were not available from all regions of Bulgaria, limiting the geographic representativeness and potentially affecting the generalizability of the findings.

## 4. Materials and Methods

### 4.1. Study Population and Epidemiological Criteria

The present study was conducted between 2022 and 2025, involving a cohort of 128 patients with laboratory-confirmed enterobiasis from 17 administrative regions of Bulgaria. Participants were enrolled following clinical indications or detection during routine prophylactic screenings. For each subject, comprehensive socio-demographic data (age, gender, residence, and education level), clinical symptomatology, and history of previous parasitic infections and treatments were documented via a structured questionnaire. The study adhered to international ethical standards, ensuring the complete anonymization of patient data, and approved by the Institutional Review Board (IRB 00006384) of the National Centre of Infectious and Parasitic Diseases (protocol #5/29 May 2024).

### 4.2. Parasitological Diagnosis and Sample Pre-Treatment

Diagnosis was established via microscopic examination of perianal impressions (transparent adhesive tape method) collected early in the morning. Each patient underwent a triple examination protocol, to ensure study specificity. Concurrent infections with other intestinal protozoa or helminths were excluded through microscopic analysis of both adhesive tape and fecal samples.

For molecular analysis parasite eggs were recovered from the glass slides using a disposable scalpel and transferred into tubes containing 200 µL of sterile saline solution. Samples were stored at −20 °C prior to processing. To optimize DNA yield, samples were subjected to a specialized pre-treatment consisting of three successive freeze–thaw cycles (freezing at −80 °C and thawing at room temperature). This procedure was previously validated by our team to enhance the extraction of parasitic DNA through the mechanical disruption of the resistant egg shells [40].

### 4.3. DNA Extraction and PCR Amplification

Total genomic DNA was isolated using the PureLink Genomic DNA Mini Kit (Invitrogen Corporation, 1600 Faraday Ave, Carlsbad, CA 92008, USA) according to the manufacturer’s instructions. Specifically, 30 µL of Proteinase K were added to 200 µL of ATL Digestion buffer and incubated at 56 °C for 24 h. Purified DNA was eluted in 100 µL of Elution buffer and stored at −20 °C.

Amplification of a fragment of the mitochondrial cytochrome c oxidase subunit 1 (*cox1*) gene was performed using a nested PCR protocol based on the method by Piperaki et al. 2011 [16]. The primer sequences were as follows:

- Outer PCR: EVM1 (Forward: 5′-TTTTTGGTCATCCTGAGGTTTATATTC-3′) and EVM2 (Reverse: 5′-CCACATTATCCAAAATAGGATTAGCC-3′), yielding a ~390 bp product.
- Inner PCR: EVIF (Forward: 5′-TTGGTCATCCTGAGGTTTATATTC-3′) and EVIR (Reverse: 5′-TCCAAAATAGGATTAGCCAACA-3′), yielding a ~379 bp product.

PCR reactions were performed in 25-µL volumes using AmpliTaq Gold 360 Master Mix (Applied Biosystems/Thermo Fisher Scientific, Foster City, CA, USA), GC Enhancer, 0.2 mM primers, and 3 µL of template DNA. Thermal cycling (BIOER Gene Explorer, Hangzhou Bioer Technology Co., Ltd., Hangzhou, China) for the first round included: initial denaturation at 95 °C for 10 min; 45 cycles of 95 °C (1 min); 57 °C (1 min); and 72 °C (1 min); and a final extension at 72 °C (10 min). The second round (using 3 µL of the first-round product) involved initial denaturation at 95 °C for 5 min, an annealing temperature of 53 °C, and 30 cycles. To ensure accurate results and detect possible contamination when performing nested PCR for the molecular biological determination of *E. vermicularis*, a negative control was included in each PCR reaction (which tested six to eight patients). Amplification of the expected gene was never detected in any of the negative controls, and a negative result was always reported. This methodology was validated for Bulgarian isolates by our team [40].

### 4.4. Electrophoresis and Sequencing Analysis

Amplicons (8 µL) were separated on a 3% agarose gel containing 0.5 µg/mL peqGREEN DNA/RNA dye (VWR International, Radnor, PA, USA). Results were visualized on a UV-transilluminator and documented using the SYNGENE system (GelVue Model No. GVM20. Synoptics Ltd., Cambridge, UK). Molecular size was determined using the 100 bp Gene Ruler™ DNA Ladder (Thermo Fisher Scientific, Waltham, MA, USA).

PCR products were purified enzymatically using the PCR Clean-up reagent (Thermo Fisher Scientific, Waltham, MA, USA) containing Exonuclease I and Alkaline Phosphatase (37 °C for 15 min. followed by inactivation at 80 °C for 15 min). Cycle sequencing was performed with the BigDye™ Terminator v3.1 Kit (Applied Biosystems) using the following profile: 96 °C for 1 min; 25 cycles of 96 °C (10 s); 50 °C (5 s); and 60 °C (4 min). Products were purified via sodium acetate/ethanol/EDTA precipitation, resuspended in 20 μL Hi-Di™ Formamide, and denatured at 95 °C for 2 min. Capillary electrophoresis was conducted on the Applied Biosystems 3500xL Genetic Analyser.

### 4.5. Bioinformatics and Phylogenetic Reconstruction

Sequence data were processed and analyzed using Geneious Prime v2021.1.1 (https://www.geneious.com) to verify quality and confirm base calls. Ambiguous nucleotide positions (R. Y. W. S. K. M) were conservatively coded as “N” (unknown) to maintain the integrity of the dataset and prevent artificial variation.

A total of 196 sequences from Bulgarian isolates were generated and deposited in GenBank (accession numbers PX210552–PX210747). To avoid redundancy in the phylogenetic analysis, 128 representative consensus sequences were selected based on the exclusion of identical sequences and retention of unique haplotypes only.

The 128 Bulgarian consensus sequences correspond to the following Accession Numbers: PX210552-PX210559; PX210561; PX210562; PX210564-PX210586; PX210588; PX210589; PX210591-PX210599; PX210603; PX210606; PX210607; PX210609-PX210611; PX210613; PX210615-PX210617; PX210621; PX210622; PX210625; PX210626; PX210629; PX210637; PX210638; PX210640; PX210641; PX210644; PX210645; PX210647; PX210648; PX210650; PX210655-PX210657; PX210659-PX210667; PX210669; PX210670; PX210672; PX210675; PX210677; PX210679; PX210680; PX210686; PX210687-PX210695; PX210698; PX210700; PX210701; PX210703; PX210704; PX210711; PX210713; PX210716; PX210717; PX210721; PX210726-PX210728; PX210730-PX210747.

Reference sequences, together with closely related sequences identified through BLAST+2.17.0 searches and publicly available sequences from countries neighboring Bulgaria (n = 116), were retrieved from GenBank, representing a broad global distribution (e.g., Iran, Japan, Denmark, Thailand, Iraq, Greece, Turkey) (Table S1). Multiple sequence alignment (n = 244; 324 bp) was performed using the MUSCLE algorithm [41] as implemented in AliView v1.17.1 [42]. Phylogenetic reconstruction was carried out using the Maximum Likelihood (ML) approach implemented in the IQ-TREE v1.6.12 web server [43]. The dataset comprised 244 *E. vermicularis cox1* sequences, including 128 newly generated Bulgarian sequences and 116 reference sequences representing global genetic diversity. The best-fitting nucleotide substitution model (TN+F+G4) was selected according to the Bayesian Information Criterion (BIC) using ModelFinder [44]. Branch support was assessed with 1000 ultrafast bootstrap replicates. The resulting phylogenetic tree was midpoint-rooted and visualized using FigTree v1.4.4, following the methodology described in our previous study [45].

Region abbreviations used in the sequence identifiers are as follows: BLA: Blagoevgrad; BOT: Botevgrad; CSO: Conevo; DC: Dolni Chiflik; DOB: Dobrich; EP: Elin Pelin; GD: Gotse Delchev; HAR: Harmanli; IHT: Ihtiman; KAR: Karlovo; KAV: Kavarna; KOS: Kostinbrod; KRU: Krumovgrad; MEZ: Mezdra; MON: Montana; NH: Novi Han; PER: Pernik; PET: Petrich; PIR: Pirdop; PLE: Pleven; PRO: Provadia; RAD: Radomir; RAZ: Razgrad; SHU: Shumen; SIL: Silistra; SOF: Sofia; TET: Teteven; TRO: Troyan; VAR: Varna; VARSH: Varchets; VRA: Vratsa; YAM: Yambol.

### 4.6. Haplotype and Genetic Diversity

Genetic diversity within the analyzed dataset was assessed using standard population genetic indices, including haplotype diversity (Hd), nucleotide diversity (π), and Tajima’s D, as implemented in DnaSP v6.12.03 [46]. Haplotype diversity (Hd) quantifies the probability that two randomly selected sequences represent different haplotypes and thus reflects the overall genetic variability and genealogical structure of the population. Nucleotide diversity (π) represents the average number of nucleotide differences per site between all possible pairs of sequences, providing a measure of sequence-level genetic variation. Tajima’s D neutrality test was applied to evaluate deviations from the neutral model of molecular evolution, with significantly positive or negative values potentially indicating demographic events (e.g., population bottlenecks or expansions) or the influence of natural selection.

Alignment positions containing gaps or missing data were excluded using the “Complete deletion” option, resulting in a final dataset of 292 nucleotide sites used for downstream analyses. Statistical significance for all population genetic tests was evaluated at a threshold of < 0.05.

### 4.7. Statistical Analysis

Statistical analyses were performed using standard statistical software. The choice of statistical tests for each comparison was based on the distribution and sample size of the data. Descriptive statistics were applied to summarize demographic and epidemiological characteristics of the study population. Frequencies and percentages were calculated for categorical variables, including gender, age groups, and geographic regions, with proportions reported alongside 95% confidence intervals (CI) estimated using the exact Clopper–Pearson method.

Associations between categorical variables were assessed using appropriate inferential tests, including the Pearson chi-square (*χ*^2^) test and Fisher’s exact test when expected cell counts were below five, particularly for comparisons between Sofia City (the capital of Bulgaria) and other regions. All statistical analyses were two-tailed, and a value < 0.05 was considered statistically significant.

## 5. Conclusions

This study offers the first comprehensive molecular characterization of *E. vermicularis* in Bulgaria, showing that the parasite population is highly homogeneous and dominated by a single genotype. Transmission appears to be widespread across the country, with young children identified as the most affected demographic group. These findings provide a foundation for evidence-based control measures and emphasize the value of ongoing molecular surveillance. Future investigations including temporal and regional comparisons will help to better understand parasite transmission dynamics and inform targeted public health strategies.

## Figures and Tables

**Table 1:** Demographic characteristics and geographical distribution of participants in the study.

Characteristic	Number	%	95% CI
Gender			
Male	62	48.4	39.5–57.4%
Female	66	51.6	42.6–60.5%
Age (years)			
Children and adolescents	118	92.2	86.2–96.2%
1–3 years	14	10.9	6.1–17.7%
4–6 years	53	41.4	32.8–50.4%
7–9 years	30	23.4	16.4–31.7%
10–12 years	19	14.8	9.2–22.2%
13–15 years	0	0.0	-
16–18 years	2	1.6	0.2–5.5%
Adults (≥19 years)	10	7.8	3.8–13.8%
Top Geographic Regions			
Sofia City	67	52.3%	43.3–61.2%
Sofia District	14	10.9%	6.1–17.7%
Other (15 regions)	47	36.8%	28.5–45.7%

**Table 2:** Haplotype distribution of *E. vermicularis* in Bulgarian populations.

Haplotype	N of Samples	Frequency (%)	Geographic Distribution
Hap_1	118	92.2	All studied regions
Hap_2	1	0.78	Ihtiman (IHT)
Hap_3	1	0.78	Pernik (PER)
Hap_4	1	0.78	Pernik (PER)
Hap_5	1	0.78	Pirdop (PIR)
Hap_6	1	0.78	Sofia (SOF)
Hap_7	1	0.78	Sofia (SOF)
Hap_8	1	0.78	Sofia (SOF)
Hap_9	1	0.78	Sofia (SOF)
Hap_10	1	0.78	Sofia (SOF)
Hap_11	1	0.78	Troyan (TRO)
Total	128	100	

**Table 3:** Summary of genetic diversity indices for *E. vermicularis cox1* sequences from Bulgaria.

Parameter	Value
Number of sequences	128
Alignment length (bp)	324
Sites analyzed (excluding gaps)	292
Polymorphic sites	13 (4.5)
Number of haplotypes	11
Haplotype diversity (Hd ± SD)	0.1507 ± 0.0416
Nucleotide diversity (π ± SD)	0.00082 ± 0.00015
Tajima’s D	−2.314 *
Dominant haplotype frequency	92.2%

* <0.05; Hd: haplotype diversity; π: nucleotide diversity; SD: standard deviation. Genetic diversity indices calculated from 128 *E. vermicularis cox1* sequences (324 bp) from Bulgaria. Sites containing gaps or missing data were excluded from the analysis using complete deletion option in DnaSP v6.12.03. Singleton variable sites represent mutations appearing only once in the dataset. Tajima’s D and Fu’s Fs statistics test for deviation from neutral evolution; negative values indicate population expansion or purifying selection. Statistical significance was assessed using 10.000 coalescent simulations.

## Data Availability

The original contributions presented in this study are included in the article. Further inquiries can be directed to the corresponding author.

## Supplementary Materials

The following supporting information can be downloaded at: https://www.mdpi.com/article/10.3390/ijms27042020/s1.

## Abbreviations

The following abbreviations are used in this manuscript:

BIC	Bayesian Information Criterion
BLA	Blagoevgrad
BOT	Botevgrad
bp	base pair
CI	confidence interval
CSO	Conevo
DC	Dolni Chiflik
DNA	Deoxyribonucleic Acid
DOB	Dobrich
EP	Elin Pelin
GD	Gotse Delchev
HAR	Harmanli
Hd	haplotype diversity
IHT	Ihtiman
KAR	Karlovo
KAV	Kavarna
KOS	Kostinbrod
KRU	Krumovgrad
MEZ	Mezdra
MON	Montana
NH	Novi Han
PCR	polymerase chain reaction
PER	Pernik
PET	Petrich
PIR	Pirdop
PLE	Pleven
PRO	Provadia
RAD	Radomir
RAZ	Razgrad
rDNA	ribosomal Deoxyribonucleic Acid
rRNA	ribosomal Ribonucleic Acid
SD	standard deviation
SHU	Shumen
SIL	Silistra
SOF	Sofia
TET	Teteven
TRO	Troyan
VAR	Varna
VARSH	Varchets
VRA	Vratsa
YAM	Yambol
π	nucleotide diversity
