# Supplementary material for: Genotypic Analysis of Enterobius vermicularis (Rhabditida: Oxyuridae, Linnaeus, 1758) Among Infected Individuals in Bulgaria: A First Phylogenetic Study
Source: Int J Mol Sci. 2026 Feb 20;27(4):2020. doi: 10.3390/ijms27042020 (PMC12940231; doi:10.3390/ijms27042020)
Supplement: Supplementary file 1 [file ijms-27-02020-s001.zip › ijms-4116984-supplementary.pdf]

**Supplementary Table S1**. All 116 reference sequences used in this study.

| № | Species | Host | Accession number | Sequence type | Country | Database |
|---|---|---|---|---|---|---|
| 1 | *Enterobius vermicularis* | Pan troglodytes | AB221457.1 | mitochondrial CO1 gene for cytochrome oxydase subunit 1 | Japan | GenBank |
| 2 | *Enterobius vermicularis* | Pan troglodytes | AB221458.1 | mitochondrial CO1 gene for cytochrome oxydase subunit 1 | Japan | GenBank |
| 3 | *Enterobius vermicularis* | Pan troglodytes | AB221461.1 | mitochondrial CO1 gene for cytochrome oxydase subunit 1 | Japan | GenBank |
| 4 | *Enterobius vermicularis* | Pan troglodytes | AB221462.1 | mitochondrial CO1 gene for cytochrome oxydase subunit 1 | Japan | GenBank |
| 5 | *Enterobius vermicularis* | Pan troglodytes | AB221464.1 | mitochondrial CO1 gene for cytochrome oxydase subunit 1 | Japan | GenBank |
| 6 | *Enterobius vermicularis* | Pan troglodytes | AB221465.1 | mitochondrial CO1 gene for cytochrome oxydase subunit 1 | Japan | GenBank |
| 7 | *Enterobius vermicularis* | Pan troglodytes | AB221466.1 | mitochondrial CO1 gene for cytochrome oxydase subunit 1 | Japan | GenBank |
| 8 | *Enterobius vermicularis* | Pan troglodytes | AB221473.1 | mitochondrial CO1 gene for cytochrome oxydase subunit 1 | Japan | GenBank |
| 9 | *Enterobius vermicularis* | Pan troglodytes | AB221474.1 | mitochondrial CO1 gene for cytochrome oxydase subunit 1 | Japan | GenBank |
| 10 | *Enterobius vermicularis* | Pan troglodytes | AB626861.1 | mitochondrial CO1 gene for cytochrome oxydase subunit 1 | Japan | GenBank |
| 11 | *Enterobius vermicularis* | Pan troglodytes | AB626862.1 | mitochondrial CO1 gene for cytochrome oxydase subunit 1 | Japan | GenBank |
| 12 | *Enterobius vermicularis* | Pan troglodytes | AB626870.1 | mitochondrial CO1 gene for cytochrome oxydase subunit 1 | Japan | GenBank |
| 13 | *Enterobius vermicularis* | Pan troglodytes | AB626872.1 | mitochondrial CO1 gene for cytochrome oxydase subunit 1 | Japan | GenBank |
| 14 | *Enterobius vermicularis* | Pan troglodytes | AB626873.1 | mitochondrial CO1 gene for cytochrome oxydase subunit 1 | Japan | GenBank |
| 15 | *Enterobius vermicularis* | Pan troglodytes | AB626880.1 | mitochondrial CO1 gene for cytochrome oxydase subunit 1 | Japan | GenBank |
| 16 | *Enterobius vermicularis* | Pan troglodytes | AB971673.1 | mitochondrial CO1 gene for cytochrome oxydase subunit 1 | Japan | GenBank |
| 17 | *Enterobius vermicularis* | Pan troglodytes | AB971674.1 | mitochondrial CO1 gene for cytochrome oxydase subunit 1 | Japan | GenBank |
| 18 | *Enterobius vermicularis* | Homo sapiens | AB221470.1 | mitochondrial CO1 gene for cytochrome oxydase subunit 1 | Japan | GenBank |
| 19 | *Enterobius vermicularis* | Homo sapiens | AB221472.1 | mitochondrial CO1 gene for cytochrome oxydase subunit 1 | Japan | GenBank |
| 20 | *Enterobius vermicularis* | Homo sapiens | AB626864.1 | mitochondrial CO1 gene for cytochrome oxydase subunit 1 | Japan | GenBank |
| 21 | *Enterobius vermicularis* | Homo sapiens | AB626865.1 | mitochondrial CO1 gene for cytochrome oxydase subunit 1 | Japan | GenBank |
| 22 | *Enterobius vermicularis* | Homo sapiens | AB626866.1 | mitochondrial CO1 gene for cytochrome oxydase subunit 1 | Japan | GenBank |
| 23 | *Enterobius vermicularis* | Homo sapiens | AB626867.1 | mitochondrial CO1 gene for cytochrome oxydase subunit 1 | Japan | GenBank |

| | | | | | | |
|---|---|---|---|---|---|---|
| 24 | *Enterobius vermicularis* | Homo sapiens | AB626868.1 | mitochondrial CO1 gene for cytochrome oxydase subunit 1 | Japan | GenBank |
| 25 | *Enterobius vermicularis* | Homo sapiens | AB626869.1 | mitochondrial CO1 gene for cytochrome oxydase subunit 1 | Japan | GenBank |
| 26 | *Enterobius vermicularis* | Homo sapiens | AB626863.1 | mitochondrial CO1 gene for cytochrome oxydase subunit 1 | Sudan | GenBank |
| 27 | *Enterobius vermicularis* | Homo sapiens | AB626871.1 | mitochondrial CO1 gene for cytochrome oxydase subunit 1 | Czech Republic | GenBank |
| 28 | *Enterobius vermicularis* | Homo sapiens | FR687965.1 | mitochondrial CO1 gene for cytochrome oxydase subunit 1 | Czech Republic | GenBank |
| 29 | *Enterobius vermicularis* | Homo sapiens | EU281143.1 | mitochondrial CO1 gene for cytochrome oxydase subunit 1 | South Korea | GenBank |
| 30 | *Enterobius vermicularis* | Homo sapiens | HQ317429.1 | mitochondrial CO1 gene for cytochrome oxydase subunit 1 | Greece | GenBank |
| 31 | *Enterobius vermicularis* | Homo sapiens | HQ317430.1 | mitochondrial CO1 gene for cytochrome oxydase subunit 1 | Greece | GenBank |
| 32 | *Enterobius vermicularis* | Homo sapiens | HQ317434.1 | mitochondrial CO1 gene for cytochrome oxydase subunit 1 | Greece | GenBank |
| 33 | *Enterobius vermicularis* | Homo sapiens | HQ317435.1 | mitochondrial CO1 gene for cytochrome oxydase subunit 1 | Greece | GenBank |
| 34 | *Enterobius vermicularis* | Homo sapiens | HQ317438.1 | mitochondrial CO1 gene for cytochrome oxydase subunit 1 | Greece | GenBank |
| 35 | *Enterobius vermicularis* | Homo sapiens | HQ317439.1 | mitochondrial CO1 gene for cytochrome oxydase subunit 1 | Greece | GenBank |
| 36 | *Enterobius vermicularis* | Homo sapiens | HQ317440.1 | mitochondrial CO1 gene for cytochrome oxydase subunit 1 | Greece | GenBank |
| 37 | *Enterobius vermicularis* | Homo sapiens | HQ395270.1 | mitochondrial CO1 gene for cytochrome oxydase subunit 1 | Greece | GenBank |
| 38 | *Enterobius vermicularis* | Homo sapiens | HQ395271.1 | mitochondrial CO1 gene for cytochrome oxydase subunit 1 | Greece | GenBank |
| 39 | *Enterobius vermicularis* | Homo sapiens | JQ411483.1 | mitochondrial CO1 gene for cytochrome oxydase subunit 1 | Denmark | GenBank |
| 40 | *Enterobius vermicularis* | Homo sapiens | JQ411484.1 | mitochondrial CO1 gene for cytochrome oxydase subunit 1 | Denmark | GenBank |
| 41 | *Enterobius vermicularis* | Homo sapiens | JQ411489.1 | mitochondrial CO1 gene for cytochrome oxydase subunit 1 | Denmark | GenBank |
| 42 | *Enterobius vermicularis* | Homo sapiens | JQ411490.1 | mitochondrial CO1 gene for cytochrome oxydase subunit 1 | Denmark | GenBank |
| 43 | *Enterobius vermicularis* | Homo sapiens | JQ411491.1 | mitochondrial CO1 gene for cytochrome oxydase subunit 1 | Denmark | GenBank |
| 44 | *Enterobius vermicularis* | Homo sapiens | JQ411492.1 | mitochondrial CO1 gene for cytochrome oxydase subunit 1 | Denmark | GenBank |
| 45 | *Enterobius vermicularis* | Homo sapiens | JQ411495.1 | mitochondrial CO1 gene for cytochrome oxydase subunit 1 | Denmark | GenBank |
| 46 | *Enterobius vermicularis* | Homo sapiens | JQ411496.1 | mitochondrial CO1 gene for cytochrome oxydase subunit 1 | Denmark | GenBank |
| 47 | *Enterobius vermicularis* | Homo sapiens | JQ411497.1 | mitochondrial CO1 gene for cytochrome oxydase subunit 1 | Denmark | GenBank |
| 48 | *Enterobius vermicularis* | Homo sapiens | JQ411502.1 | mitochondrial CO1 gene for cytochrome oxydase subunit 1 | Denmark | GenBank |

| | | | | | | |
|---|---|---|---|---|---|---|
| 49 | *Enterobius vermicularis* | Homo sapiens | JQ411504.1 | mitochondrial CO1 gene for cytochrome oxydase subunit 1 | Denmark | GenBank |
| 50 | *Enterobius vermicularis* | Homo sapiens | JQ411508.1 | mitochondrial CO1 gene for cytochrome oxydase subunit 1 | Denmark | GenBank |
| 51 | *Enterobius vermicularis* | Homo sapiens | JQ411509.1 | mitochondrial CO1 gene for cytochrome oxydase subunit 1 | Denmark | GenBank |
| 52 | *Enterobius vermicularis* | Homo sapiens | JQ411505.1 | mitochondrial CO1 gene for cytochrome oxydase subunit 1 | Germany | GenBank |
| 53 | *Enterobius vermicularis* | Homo sapiens | KX527600.1 | mitochondrial CO1 gene for cytochrome oxydase subunit 1 | Poland | GenBank |
| 54 | *Enterobius vermicularis* | Homo sapiens | KX527601.1 | mitochondrial CO1 gene for cytochrome oxydase subunit 1 | Poland | GenBank |
| 55 | *Enterobius vermicularis* | Homo sapiens | KX527602.1 | mitochondrial CO1 gene for cytochrome oxydase subunit 1 | Poland | GenBank |
| 56 | *Enterobius vermicularis* | Homo sapiens | KJ780776.1 | mitochondrial CO1 gene for cytochrome oxydase subunit 1 | Iran (Tabriz) | GenBank |
| 57 | *Enterobius vermicularis* | Homo sapiens | KJ780777.1 | mitochondrial CO1 gene for cytochrome oxydase subunit 1 | Iran (Tabriz) | GenBank |
| 58 | *Enterobius vermicularis* | Homo sapiens | LC819589.1 | mitochondrial CO1 gene for cytochrome oxydase subunit 1 | Iraq (Mosul) | GenBank |
| 59 | *Enterobius vermicularis* | Homo sapiens | LC819590.1 | mitochondrial CO1 gene for cytochrome oxydase subunit 1 | Iraq (Mosul) | GenBank |
| 60 | *Enterobius vermicularis* | Homo sapiens | LC819591.1 | mitochondrial CO1 gene for cytochrome oxydase subunit 1 | Iraq (Mosul) | GenBank |
| 61 | *Enterobius vermicularis* | Homo sapiens | LC819593.1 | mitochondrial CO1 gene for cytochrome oxydase subunit 1 | Iraq (Mosul) | GenBank |
| 62 | *Enterobius vermicularis* | Homo sapiens | LC819596.1 | mitochondrial CO1 gene for cytochrome oxydase subunit 1 | Iraq (Mosul) | GenBank |
| 63 | *Enterobius vermicularis* | Homo sapiens | LC819597.1 | mitochondrial CO1 gene for cytochrome oxydase subunit 1 | Iraq (Mosul) | GenBank |
| 64 | *Enterobius vermicularis* | Homo sapiens | LC819598.1 | mitochondrial CO1 gene for cytochrome oxydase subunit 1 | Iraq (Mosul) | GenBank |
| 65 | *Enterobius vermicularis* | Homo sapiens | LC819599.1 | mitochondrial CO1 gene for cytochrome oxydase subunit 1 | Iraq (Mosul) | GenBank |
| 66 | *Enterobius vermicularis* | Homo sapiens | LC819600.1 | mitochondrial CO1 gene for cytochrome oxydase subunit 1 | Iraq (Mosul) | GenBank |
| 67 | *Enterobius vermicularis* | Homo sapiens | LC819601.1 | mitochondrial CO1 gene for cytochrome oxydase subunit 1 | Iraq (Mosul) | GenBank |
| 68 | *Enterobius vermicularis* | Homo sapiens | MH208464.1 | mitochondrial CO1 gene for cytochrome oxydase subunit 1 | Thailand | GenBank |
| 69 | *Enterobius vermicularis* | Homo sapiens | MH208467.1 | mitochondrial CO1 gene for cytochrome oxydase subunit 1 | Thailand | GenBank |
| 70 | *Enterobius vermicularis* | Homo sapiens | MH208468.1 | mitochondrial CO1 gene for cytochrome oxydase subunit 1 | Thailand | GenBank |
| 71 | *Enterobius vermicularis* | Homo sapiens | MH208469.1 | mitochondrial CO1 gene for cytochrome oxydase subunit 1 | Thailand | GenBank |
| 72 | *Enterobius vermicularis* | Homo sapiens | MH208470.1 | mitochondrial CO1 gene for cytochrome oxydase subunit 1 | Thailand | GenBank |
| 73 | *Enterobius vermicularis* | Homo sapiens | MH208473.1 | mitochondrial CO1 gene for cytochrome oxydase subunit 1 | Thailand | GenBank |

| | | | | | | |
|---|---|---|---|---|---|---|
| 74 | *Enterobius vermicularis* | Homo sapiens | MH208474.1 | mitochondrial CO1 gene for cytochrome oxydase subunit 1 | Thailand | GenBank |
| 75 | *Enterobius vermicularis* | Homo sapiens | MH208475.1 | mitochondrial CO1 gene for cytochrome oxydase subunit 1 | Thailand | GenBank |
| 76 | *Enterobius vermicularis* | Homo sapiens | MH208476.1 | mitochondrial CO1 gene for cytochrome oxydase subunit 1 | Thailand | GenBank |
| 77 | *Enterobius vermicularis* | Homo sapiens | MH208477.1 | mitochondrial CO1 gene for cytochrome oxydase subunit 1 | Thailand | GenBank |
| 78 | *Enterobius vermicularis* | Homo sapiens | MH208478.1 | mitochondrial CO1 gene for cytochrome oxydase subunit 1 | Thailand | GenBank |
| 79 | *Enterobius vermicularis* | Homo sapiens | MH208480.1 | mitochondrial CO1 gene for cytochrome oxydase subunit 1 | Thailand | GenBank |
| 80 | *Enterobius vermicularis* | Homo sapiens | MH802591.1 | mitochondrial CO1 gene for cytochrome oxydase subunit 1 | Iran | GenBank |
| 81 | *Enterobius vermicularis* | Homo sapiens | MH802593.1 | mitochondrial CO1 gene for cytochrome oxydase subunit 1 | Iran | GenBank |
| 82 | *Enterobius vermicularis* | Homo sapiens | MH802594.1 | mitochondrial CO1 gene for cytochrome oxydase subunit 1 | Iran | GenBank |
| 83 | *Enterobius vermicularis* | Homo sapiens | MH802595.1 | mitochondrial CO1 gene for cytochrome oxydase subunit 1 | Iran | GenBank |
| 84 | *Enterobius vermicularis* | Homo sapiens | MH802596.1 | mitochondrial CO1 gene for cytochrome oxydase subunit 1 | Iran | GenBank |
| 85 | *Enterobius vermicularis* | Homo sapiens | MH802597.1 | mitochondrial CO1 gene for cytochrome oxydase subunit 1 | Iran | GenBank |
| 86 | *Enterobius vermicularis* | Homo sapiens | MH802600.1 | mitochondrial CO1 gene for cytochrome oxydase subunit 1 | Iran | GenBank |
| 87 | *Enterobius vermicularis* | Homo sapiens | MH802601.1 | mitochondrial CO1 gene for cytochrome oxydase subunit 1 | Iran | GenBank |
| 88 | *Enterobius vermicularis* | Homo sapiens | MH802602.1 | mitochondrial CO1 gene for cytochrome oxydase subunit 1 | Iran | GenBank |
| 89 | *Enterobius vermicularis* | Homo sapiens | MH802604.1 | mitochondrial CO1 gene for cytochrome oxydase subunit 1 | Iran | GenBank |
| 90 | *Enterobius vermicularis* | Homo sapiens | MH802605.1 | mitochondrial CO1 gene for cytochrome oxydase subunit 1 | Iran | GenBank |
| 91 | *Enterobius vermicularis* | Homo sapiens | MH802606.1 | mitochondrial CO1 gene for cytochrome oxydase subunit 1 | Iran | GenBank |
| 92 | *Enterobius vermicularis* | Homo sapiens | MH802607.1 | mitochondrial CO1 gene for cytochrome oxydase subunit 1 | Iran | GenBank |
| 93 | *Enterobius vermicularis* | Homo sapiens | MH802610.1 | mitochondrial CO1 gene for cytochrome oxydase subunit 1 | Iran | GenBank |
| 94 | *Enterobius vermicularis* | Homo sapiens | MH802611.1 | mitochondrial CO1 gene for cytochrome oxydase subunit 1 | Iran | GenBank |
| 95 | *Enterobius vermicularis* | Homo sapiens | MZ360956.1 | mitochondrial CO1 gene for cytochrome oxydase subunit 1 | Iran | GenBank |
| 96 | *Enterobius vermicularis* | Homo sapiens | MZ360957.1 | mitochondrial CO1 gene for cytochrome oxydase subunit 1 | Iran | GenBank |
| 97 | *Enterobius vermicularis* | Homo sapiens | MZ360958.1 | mitochondrial CO1 gene for cytochrome oxydase subunit 1 | Iran | GenBank |
| 98 | *Enterobius vermicularis* | Homo sapiens | MZ361995.1 | mitochondrial CO1 gene for cytochrome oxydase subunit 1 | Iran | GenBank |

| | | | | | | |
|---|---|---|---|---|---|---|
| 99 | *Enterobius vermicularis* | Homo sapiens | MZ361996.1 | mitochondrial CO1 gene for cytochrome oxydase subunit 1 | Iran | GenBank |
| 100 | *Enterobius vermicularis* | Homo sapiens | MZ361998.1 | mitochondrial CO1 gene for cytochrome oxydase subunit 1 | Iran | GenBank |
| 101 | *Enterobius vermicularis* | Homo sapiens | MZ361999.1 | mitochondrial CO1 gene for cytochrome oxydase subunit 1 | Iran | GenBank |
| 102 | *Enterobius vermicularis* | Homo sapiens | MZ362434.1 | mitochondrial CO1 gene for cytochrome oxydase subunit 1 | Iran | GenBank |
| 103 | *Enterobius vermicularis* | Homo sapiens | OL773355.1 | mitochondrial CO1 gene for cytochrome oxydase subunit 1 | Iran | GenBank |
| 104 | *Enterobius vermicularis* | Homo sapiens | OL773359.1 | mitochondrial CO1 gene for cytochrome oxydase subunit 1 | Iran | GenBank |
| 105 | *Enterobius vermicularis* | Homo sapiens | OL773362.1 | mitochondrial CO1 gene for cytochrome oxydase subunit 1 | Iran | GenBank |
| 106 | *Enterobius vermicularis* | Homo sapiens | PV131523.1 | mitochondrial CO1 gene for cytochrome oxydase subunit 1 | Turkey (Mersin) | GenBank |
| 107 | *Enterobius vermicularis* | Homo sapiens | PV131524.1 | mitochondrial CO1 gene for cytochrome oxydase subunit 1 | Turkey (Mersin) | GenBank |
| 108 | *Enterobius vermicularis* | Homo sapiens | PV131525.1 | mitochondrial CO1 gene for cytochrome oxydase subunit 1 | Turkey (Mersin) | GenBank |
| 109 | *Enterobius vermicularis* | Homo sapiens | PV131526.1 | mitochondrial CO1 gene for cytochrome oxydase subunit 1 | Turkey (Mersin) | GenBank |
| 110 | *Enterobius vermicularis* | Homo sapiens | PV131527.1 | mitochondrial CO1 gene for cytochrome oxydase subunit 1 | Turkey (Mersin) | GenBank |
| 111 | *Enterobius vermicularis* | Homo sapiens | PV131531.1 | mitochondrial CO1 gene for cytochrome oxydase subunit 1 | Turkey (Mersin) | GenBank |
| 112 | *Enterobius vermicularis* | Homo sapiens | PV131532.1 | mitochondrial CO1 gene for cytochrome oxydase subunit 1 | Turkey (Mersin) | GenBank |
| 113 | *Enterobius vermicularis* | Homo sapiens | PV131533.1 | mitochondrial CO1 gene for cytochrome oxydase subunit 1 | Turkey (Mersin) | GenBank |
| 114 | *Enterobius vermicularis* | Homo sapiens | PV131534.1 | mitochondrial CO1 gene for cytochrome oxydase subunit 1 | Turkey (Mersin) | GenBank |
| 115 | *Enterobius vermicularis* | Homo sapiens | AP017684.1 | mitochondrial CO1 gene for cytochrome oxydase subunit 1 | — | GenBank |
| 116 | *Enterobius vermicularis* | — | NC_056632.1 | mitochondrial CO1 gene for cytochrome oxydase subunit 1 | — | GenBank |
